# Cardiac Adaptation in Lupus: A Case of Massive Pericardial Effusion With Preserved Hemodynamics

**DOI:** 10.7759/cureus.89152

**Published:** 2025-07-31

**Authors:** Zaineb Khawar, Maria B Herrera-Gonzalez, Mariam Mirza, Noreen Mirza, Addi Suleiman

**Affiliations:** 1 Internal Medicine, Saint Michael's Medical Center, Newark, USA; 2 Medical Education, Saint Michael's Medical Center, Newark, USA; 3 Cardiology, Saint Michael's Medical Center, Newark, USA; 4 Cardiology, Saint Michael's Medical Center/New York Medical College, Newark, USA

**Keywords:** echocardiography in cardio-oncology, lupus pericarditis, massive pericardial effusion, pericardial compliance, pericardial effusion without tamponade, pericardiocentesis, systemic lupus erythema

## Abstract

Systemic lupus erythematosus (SLE) is a multisystem autoimmune disease; cardiac involvement is a recognized complication, with pericardial effusion being one of the most frequent manifestations. Here, we present a patient with massive pericardial effusion in a known SLE patient without hemodynamic instability, highlighting concepts of pericardial compliance and physiological adaptation in autoimmune disease. We present a case of a 33-year-old female with a known history of SLE who presented with progressively worsening pleuritic chest pain over three weeks. She was hemodynamically stable, with distant heart sounds and no jugular venous distension. Her initial encounter was suspicious of pulmonary embolism, and a CT angiography of the chest was performed, which revealed the true culprit of her symptoms: a large pericardial effusion. Laboratory workup showed elevated inflammatory markers and positive anti-dsNDA, RNP, and Smith antibodies. Electrocardiogram demonstrated a low voltage, but no presence of electrical alternans. Echocardiogram revealed an ejection fraction of 65%, no evidence of right ventricular diastolic collapse or tamponade, but a large pericardial effusion up to 2.7 cm. The patient was monitored in the intensive care unit, treated with corticosteroids, colchicine, and hydroxychloroquine, and underwent pericardiocentesis with drainage of over 1,100 cc of serous fluid. Her symptoms resolved, and analysis of the fluid showed no evidence of infectious or malignant etiology. Pericardial compliance refers to the pericardium's ability to stretch in response to fluid accumulation. Chronic inflammatory changes in SLE typically reduce pericardial compliance through fibrosis and pericardial thickening; a slow rate of accumulation may paradoxically permit large effusions to develop without hemodynamic compromise. This case suggests that gradual accumulation over time may allow the pericardium to adapt, delaying or preventing hemodynamic compromise. This emphasizes the interplay between effusion volume, rate of accumulation, and pericardial compliance. It is important to consider how slowly growing effusions in autoimmune diseases can behave differently and require careful monitoring and treatment planning. Persistent effusions despite immunosuppressive therapy underscore the need for better understanding and treatment strategies for this complex manifestation.

## Introduction

Systemic lupus erythematosus (SLE) is a well-known multi-systemic autoimmune disease characterized by inflammation and damage to multiple organ systems, including the joints, kidneys, and skin [1,2]. The cardiovascular system is undoubtedly one of the target organs involved in SLE and can potentially damage the pericardium, myocardium, endocardium, conduction system, and coronary arteries [3]. Pericardial effusion, which is an accumulation of excessive fluid in the pericardial sac surrounding the heart, is a common manifestation of SLE, occurring in up to 25% of patients with diagnosed lupus [1,2]. Previous studies have demonstrated that SLE is associated with an increased risk of effusion, where 1-3% of these patients are susceptible to subsequent tamponade and hemodynamic compromise [4]. We present a case of massive pericardial effusion in a known SLE patient whose case brings to light topics of pericardial compliance and adaptation in autoimmune disease.

## Case presentation

A 33-year-old female with a history of DVT and pulmonary embolism (PE) in 2014 (maintained on rivaroxaban), SLE (diagnosed in 2009, on hydroxychloroquine and belimumab), lupus nephritis, hypertension, and type 2 diabetes mellitus presented with left-sided non-radiating chest pain for three weeks that was progressively worsening. The chest pain was worse with breathing and upon lying down. Her symptoms were mildly relieved with sitting upright or leaning forward. She provided the remote history of sinus infection before the development of chest pain; however, her symptoms were not associated with fever, cough, dyspnea, nausea, or vomiting. 

**Figure 1:**
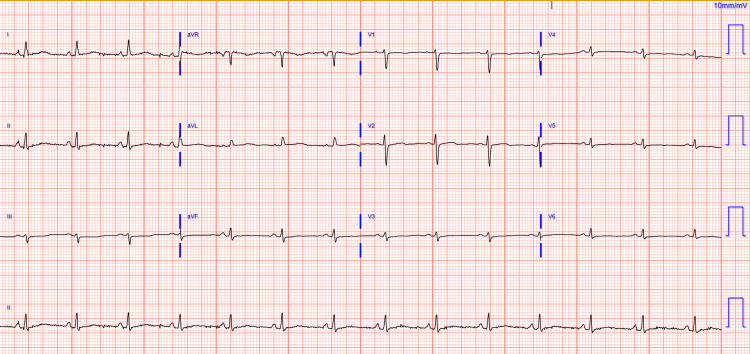
Electrocardiogram shows a low voltage, normal sinus rhythm, without acute ST wave changes or the presence of electrical alternans

**Figure 2:**
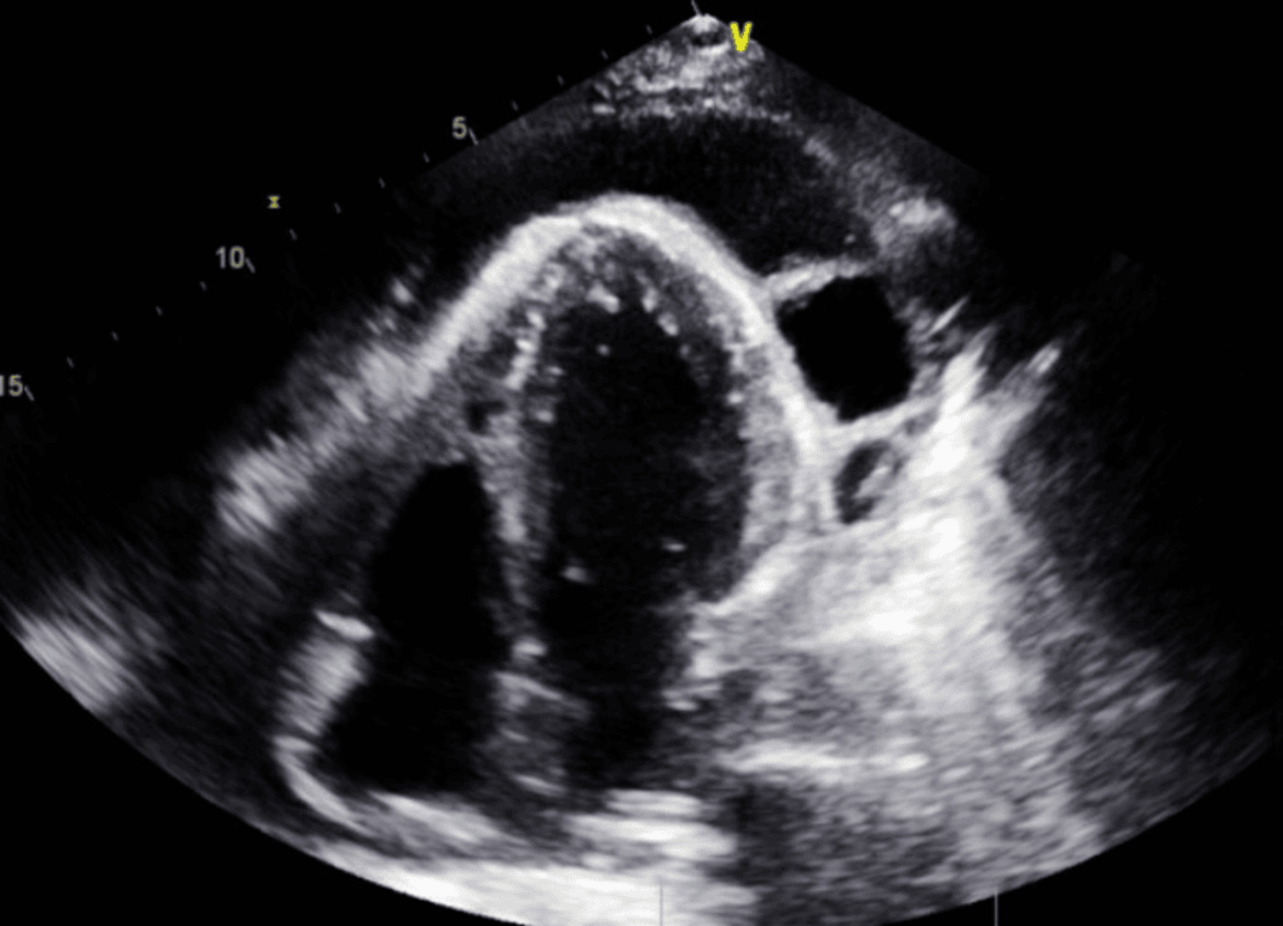
Echocardiogram on admission showing pericardial effusion

On physical examination, the patient was normotensive and hemodynamically stable. Cardiac examination was significant for distant heart sounds, otherwise a normal S1, S2, with no murmurs or pericardial rub. There was no jugular venous distension, and the lungs were clear to auscultation. There were no mucocutaneous lesions, skin rashes, or synovitis. Initial lab work showed mild anemia on CBC with a hemoglobin of 10.6 g/dL (reference: 12.0-15.5g/dL), a noncontributory BMP, an elevated INR of 3.43 (reference: 0.91-1.1, believed to be due to the patient's rivaroxaban use), and an elevated D-dimer of 3,679 ng/mL. Due to the patient's history of PE, the patient underwent CT angiography, which yielded no PE and revealed pericardial effusion with a maximum thickness of 2.5 cm. This prompted further lab investigation, which showed a negative respiratory pathogen panel, an erythrocyte sedimentation rate of 73 mm/hr, and C-reactive protein of 9.5 mg/dL. Subsequent autoimmune workup revealed anti-dsDNA 40 IU/mL with positive RNP and Smith antibodies (Table 1). Electrocardiogram demonstrated a low voltage, normal sinus rhythm, without acute ST wave changes or the presence of electrical alternans (Figure 1). An echocardiogram showed an EF of 65% and a large pericardial effusion of up to 2.7 cm with no right-ventricular diastolic collapse. There was no significant respiratory variation; however, the echocardiogram showed a fibrotic band (Figure 2).

**Table 1:** Relevant laboratory values with reference range BNP: B-type natriuretic peptide, INR: international normalized ratio, ESR: erythrocyte sedimentation rate, CRP: C-reactive protein, anti-DS DNA: anti-double-stranded DNA antibody, RNP Ab: ribonucleoprotein antibody

Laboratory Test	Laboratory Value	Reference Range
Hemoglobin	10.1 g/dL	12.0-15.5 g/dL
D-dimer	3,679 ng/mL	0.0-500 ng/mL
Troponin	<4 ng/L	<34 ng/L
BNP	130.76 pg/mL	<100 pg/mL
INR	3.43	0.91-1.1
ESR	73 mm/hr	0-20 mm/hr
CRP	9.5 mg/dL	0.0-0.8 mg/dL
Anti-DS DNA	40 IU/mL	0.0-9.0 IU/mL
Anti-Smith Ab	1.0 AI	0.0-0.9 AI
RNP Ab	>8.0 AI	0.0-0.9 AI

Given the patient's hemodynamic stability, elevated INR, and no evidence of cardiac tamponade, she was monitored in the intensive care unit. She was started on hydroxychloroquine and high-dose solumedrol dosed at 1 mg/kg with taper. Colchicine was also started for a suspected concurrent pericarditis. Pericardiocentesis was delayed for approximately 36 hours, given the elevated INR and preserved hemodynamics. INR corrected to 1.37, and the patient underwent pericardiocentesis with drain placement. The initial pericardiocentesis yielded 525 cc of serous fluid, which was followed by 600 cc of further output from the drain. Analysis of the fluid showed no evidence of infection or malignancy. Following drain removal, the patient reported resolution of her symptoms.

## Discussion

Pericardial effusion in the general population may be attributed to inciting events, including infection, inflammation, malignancy, or trauma [3]. However, in a patient with known SLE, it is essential to recognize the manifestation of pericardial effusion due to autoimmune disease processes. As previously stated, pericardial effusion is a common manifestation of SLE, occurring in up to 25% of lupus patients, with a small percentage of these patients progressing to cardiac tamponade [1,2]. The pathophysiology is thought to be caused by chronic inflammatory changes and deposition of immune complexes formed by double-stranded DNA and anti-Smith antibodies. It is theorized that these immune complexes, in addition to complement activation, contribute to increased pericardial permeability and lead to the development of pericardial effusions [3].

Diagnosis begins with the clinical manifestations of pericardial effusion, typically in a patient who presents with dyspnea, chest discomfort, and pleuritic chest pain. In patients with a past medical history of SLE, there should be a high index of suspicion for effusion and possible tamponade, particularly in the presence of hypotension. Transthoracic echocardiography (TTE) remains the gold standard for diagnosing pericardial effusion and assessing for signs of hemodynamic compromise. Echocardiography plays a critical role in the diagnosis as it would demonstrate cardiac tamponade findings such as right ventricular diastolic collapse, or inferior vena cava plethora, and warrant urgent intervention [4]. In addition to clinical findings and pertinent patient history, elevated inflammatory markers and low complement levels, which reflect active lupus, support the diagnosis. Anti-dsDNA antibodies are also typically elevated. When drained, the analysis of pericardial fluid showing a sterile, exudative fluid with antinuclear antibodies and immune complexes would support the diagnosis [4,5]. Pericardial fluid should be sent for culture, cytology, and analysis to rule out infection or malignancy. The treatment for small-to-moderate pericardial effusions is glucocorticoids and colchicine to prevent the recurrence of pericarditis. In massive, recurrent, or refractory effusions, pericardiocentesis, window placement, or pericardiectomy may be necessary [4]. Massive pericardial effusion is typically defined as an accumulation of over 500 cc when drained, and our case demonstrates an effusion with an output of over one liter. During her hospital course, the patient remained hemodynamically stable, and her echocardiography did not show evidence of cardiac tamponade, which allows this case to bring to light questions of chronic pericardial effusion leading to pericardial compliance and adaptation in SLE patients. 

Pericardial compliance refers to the ability of the pericardium to stretch and accommodate changes in cardiac volume. Classically, in SLE patients, the chronic inflammation has the converse effect on the pericardium. These patients typically experience pericardial thickening, fibrosis, and decreased compliance as a result of chronic inflammation, which subsequently results in recurrent episodes of pericarditis [5]. This further suggests that decreased compliance as an adaptation in SLE patients would put them at higher risk for cardiac tamponade at small- to moderate-sized effusions. Conversely, our case outlines how, in some cases, gradual accumulation of the effusion allows the pericardium to adapt and tolerate larger volumes before clinical manifestations [6]. The variations in adaptability demonstrate a complex relationship between effusion volume, rate of accumulation, and pericardial compliance as it relates to SLE patients. This also explains how, despite the patient’s presentation with a massive effusion, the chronic accumulation of fluid allowed for pericardial compliance and increased the threshold for cardiac tamponade [7]. 

The presence of cardiac involvement in SLE patients is thoroughly documented. Pericardial effusions can also persist despite immunosuppressive therapy, indicating the need to investigate further treatment options and bring into question the role of routine cardiac monitoring, such as scheduled follow-up echocardiograms, in SLE patients [8]. Massive pericardial effusion in SLE is an alarming presentation that requires an integrated approach between rheumatology, cardiology, and critical care. It also highlights the challenges posed by autoimmune disease and the need to further understanding, recognition, and treatment for this disease manifestation.

## Conclusions

Chronic inflammation and immune complex deposition can lead to the hallmark presentation of pericardial effusion in SLE patients. The ability of the pericardium to adapt to the progressive accumulation permits these effusions to accumulate to large volumes, sometimes without hemodynamic compromise. However, this occurs in select cases and in the instance of gradual accumulation. Understanding and recognizing this mechanism is crucial for managing SLE patients as it relates to the physiological adaptations in autoimmune disease.
